# Supplementary material for: Observation of unusual topological surface states in half-Heusler compounds LnPtBi (Ln=Lu, Y)
Source: Nat Commun. 2016 Sep 27;7:12924. doi: 10.1038/ncomms12924 (PMC5052656; doi:10.1038/ncomms12924)
Supplement: Supplementary Information — Supplementary Figures 1-10 and Supplementary Notes 1-6 [file ncomms12924-s1.pdf]

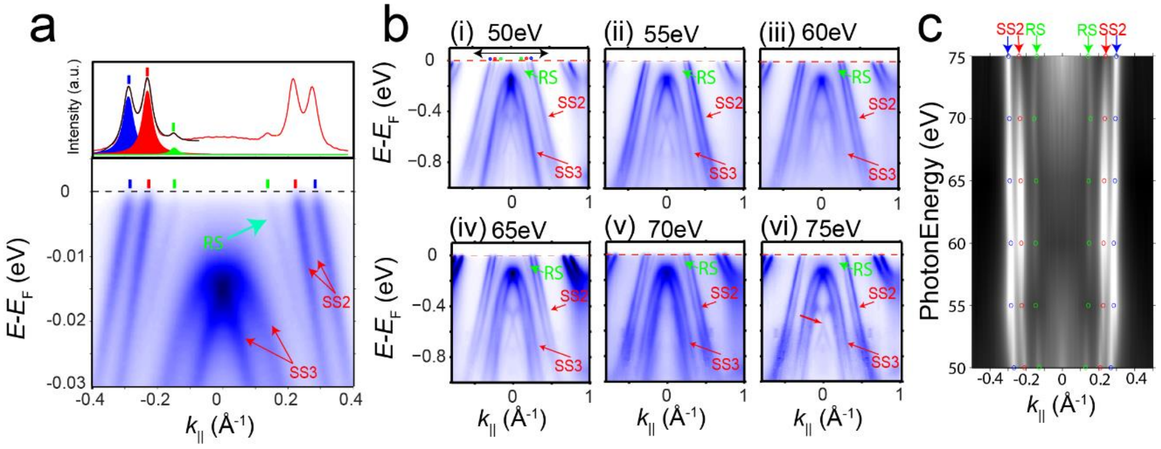

Supplementary Figure 1: **Analysis of the E_F crossing bands around $\bar{\Gamma}$. a**, Zoomed-in plot of the $\bar{\Gamma}$-$\bar{K}$ cut close to E_F. Different bands are labeled separately. The Fermi crossings are identified by fitted peaks on the MDC (the above panel). **b**, Plot of the $\bar{\Gamma}$-$\bar{K}$ cut measured at various photon energies. Different bands are labeled. **c**, Intensity plot of the stack MDCs at E_F extracted from the cuts in (**b**). For each MDC, the positions of each peaks fitted are labeled by the colored marks. SS: topologically trivial surface state. RS: surface resonant state.

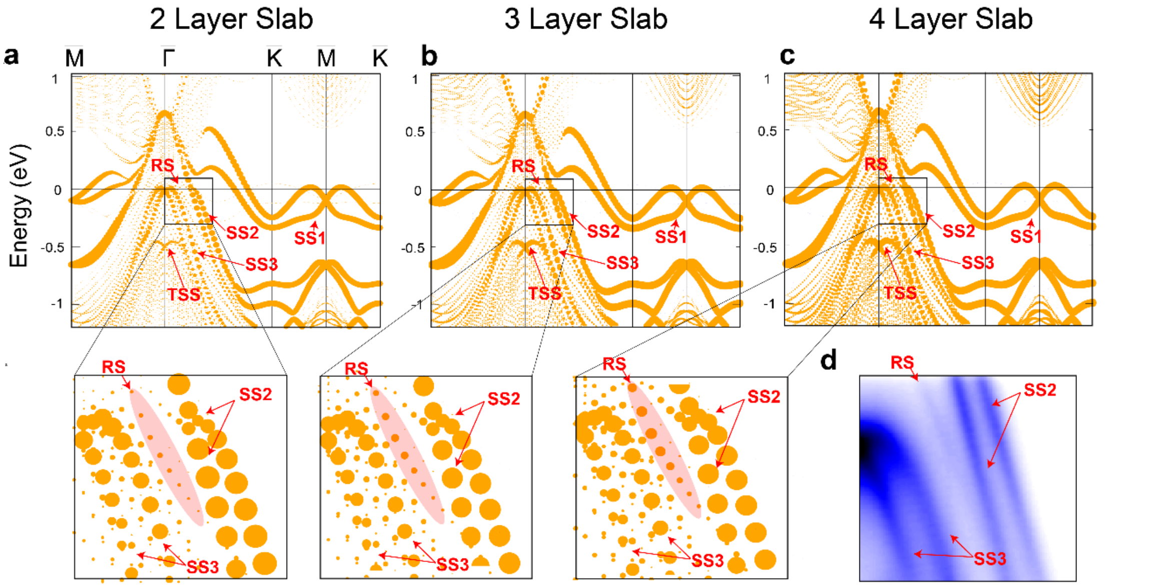

Supplementary Figure 2: **Calculated bandstructure of Bi-terminated LuPtBi (111) surface along high symmetry cuts. a-c**, Results from a slab model calculation when projected to 2-, 3- and 4- layer. The size of filled circles represent the contribution from the surface. Different bands are labeled. Zoom-in plot of the calculation results shows the details of the E_F vicinity. Different bands are labeled. Red ovals indicate the band between SS2 and SS3. **d**, Plot of the measured spectrum of the same range. Different bands are labeled. SS: topologically trivial surface state. TSS: topologically non-trivial surface state. RS: surface resonant state.

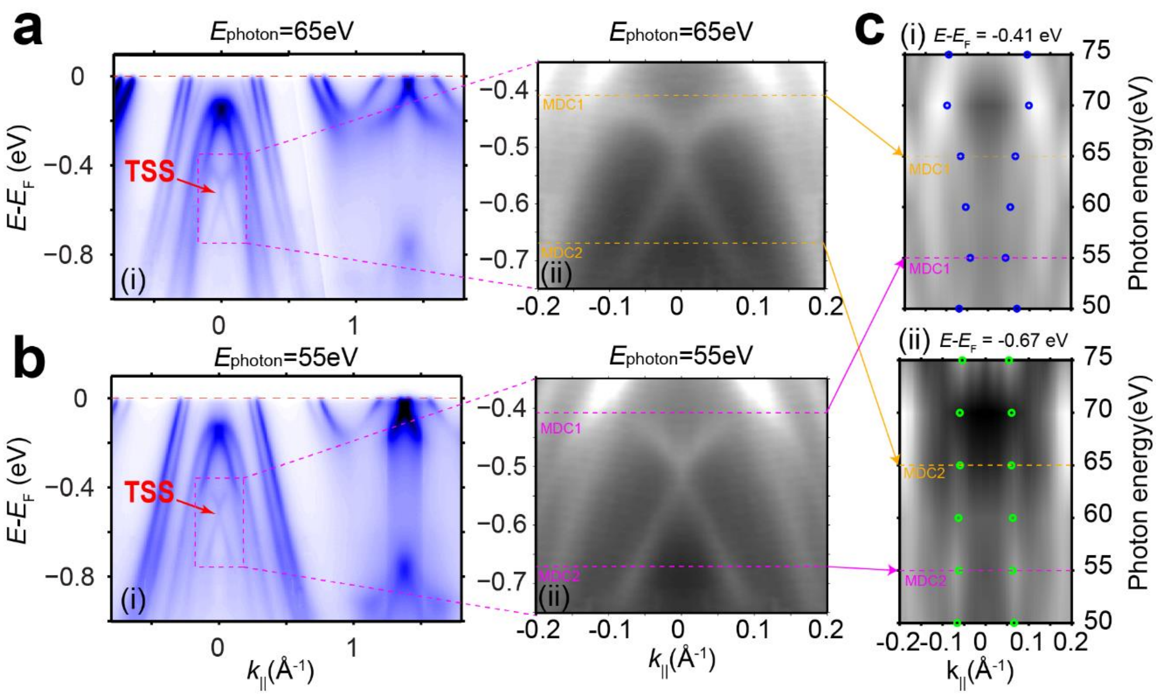

Supplementary Figure 3: **Photon energy dependence of different part of TSS. a,b,** Dispersions along high symmetry direction ($\bar{\Gamma}$-$\bar{X}$) with 65eV (**a**) and 55eV (**b**) photon energy, respectively, panels (ii) in (**a**)(**b**) show the zoom-in plots of the "X" shape TSS dispersion with details. Orange and magenta dashed lines in panels (ii) of (**a**)(**b**) indicate the two MDCs at different parts of the "X" shape dispersion which we use for photon energy dependent measurement in (**c**). **c**, Intensity plot of the photon energy dependent measurements of MDCs at 0.41eV(i) and 0.67eV(ii) binding energy. Blue and green circles represent the fitted peak positions on each of the MDC. The orange and magenta dashed lines show the MDCs from panels (ii) of (**a**) and (**b**), respectively. TSS: topological surface state.

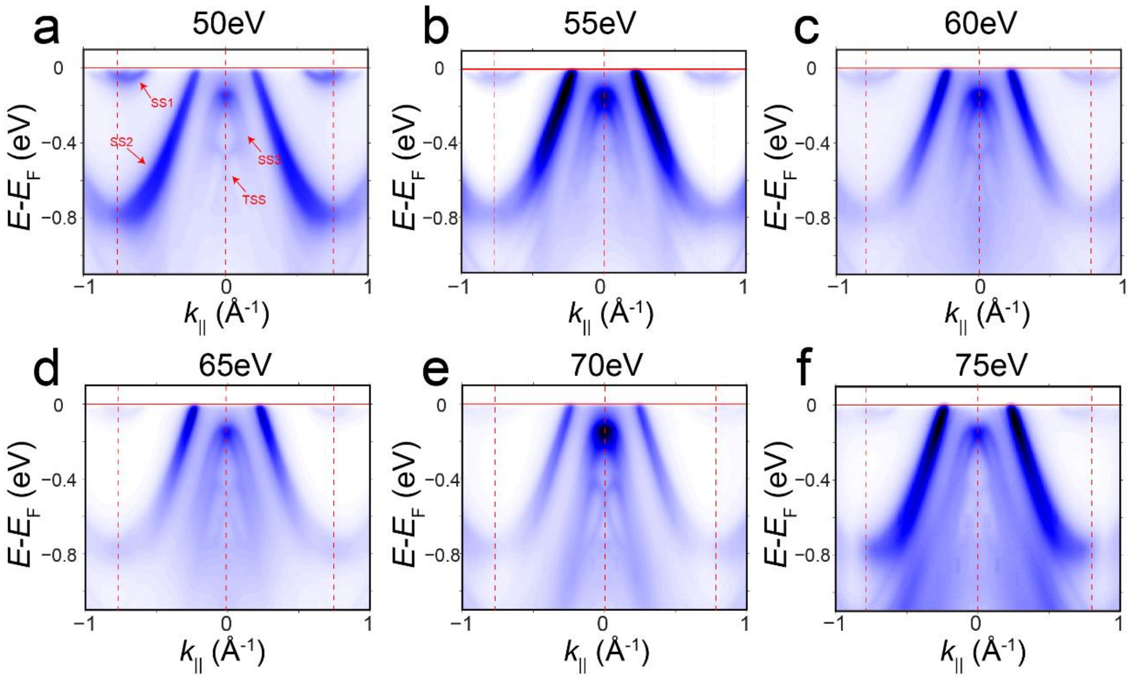

Supplementary Figure 4: **Photon energy dependence of the $\bar{\Gamma}$-$\bar{M}$ cut on the Bi-terminated, (111) surface of LuPtBi. a-f**. Photoemission intensity plot along the high symmetry $\bar{\Gamma}$-$\bar{M}$ direction with photon energies from 50 eV to 75 eV. Metallic surface states (SS1-SS3) and topological surface states (TSS) are labeled. SS: topologically trivial metallic surface state due to the dangling bonds on sample surface. TSS: topologically non-trivial surface state.

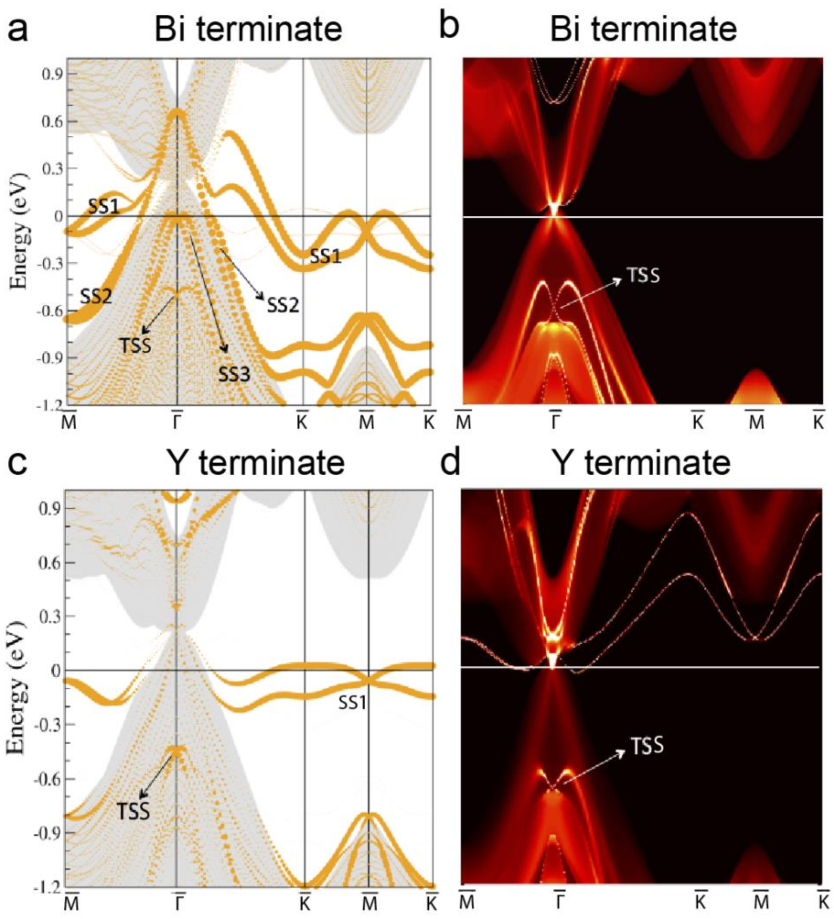

Supplementary Figure 5: **Ab-initio calculation of the electronic structure of (111) surface of YPtBi with different terminations. a-b**, Calculation results from DFT method on a slab model (**a**) and Green's function on a semi-infinite surface (**b**) on YPtBi terminated by Bi. **c-d**, Calculation results from DFT method on a slab model (**c**) and Green's function method on a semi-infinite surface (**d**) on YPtBi terminated by Y. In the slab model calculation results, the size of filled circles represent the projection to the surface. Both topologically nontrivial surface state and metallic surface states due to surface dangling bonds are captured. In the results from a semi-infinite surface, only topologically nontrivial surface state is revealed by the calculation. SS: topologically trivial metallic surface state due to the dangling bonds on sample surface. TSS: topologically non-trivial surface state.

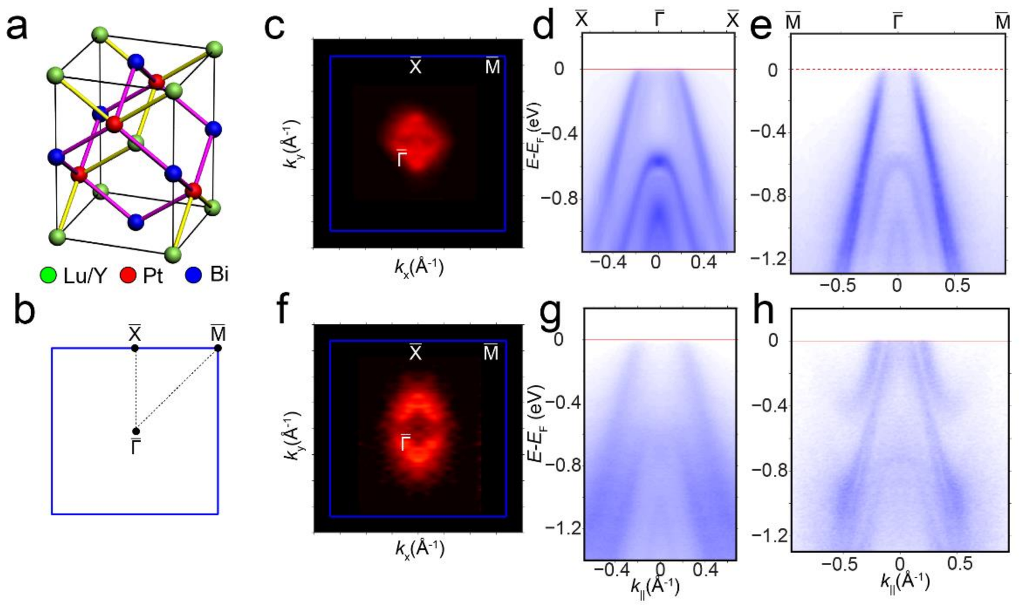

Supplementary Figure 6**: Measured electronic structure on (001) surface of YPtBi and LuPtBi.** **a**, Plot of the unit-cell on the (001) surface of YPtBi and LuPtBi. The unit-cell contains 2 atoms of each kind (Lu/Y, Pt, Bi). **b**, Derived surface BZ from the unit-cell shown in (**a**) with high symmetry points labeled. **c-e**, Fermi surface map (**c**) and high-symmetry $\bar{X}$-$\bar{\Gamma}$-$\bar{X}$ (**d**), $\bar{M}$-$\bar{\Gamma}$-$\bar{M}$ (**e**) cuts on the electronic structure on (100) surface of YPtBi. **f-h**, Fermi surface map (**f**) and high-symmetry $\bar{X}$-$\bar{\Gamma}$-$\bar{X}$ (**g**), $\bar{M}$-$\bar{\Gamma}$-$\bar{M}$ (**h**) cuts on the electronic structure on (001) surface of LuPtBi.

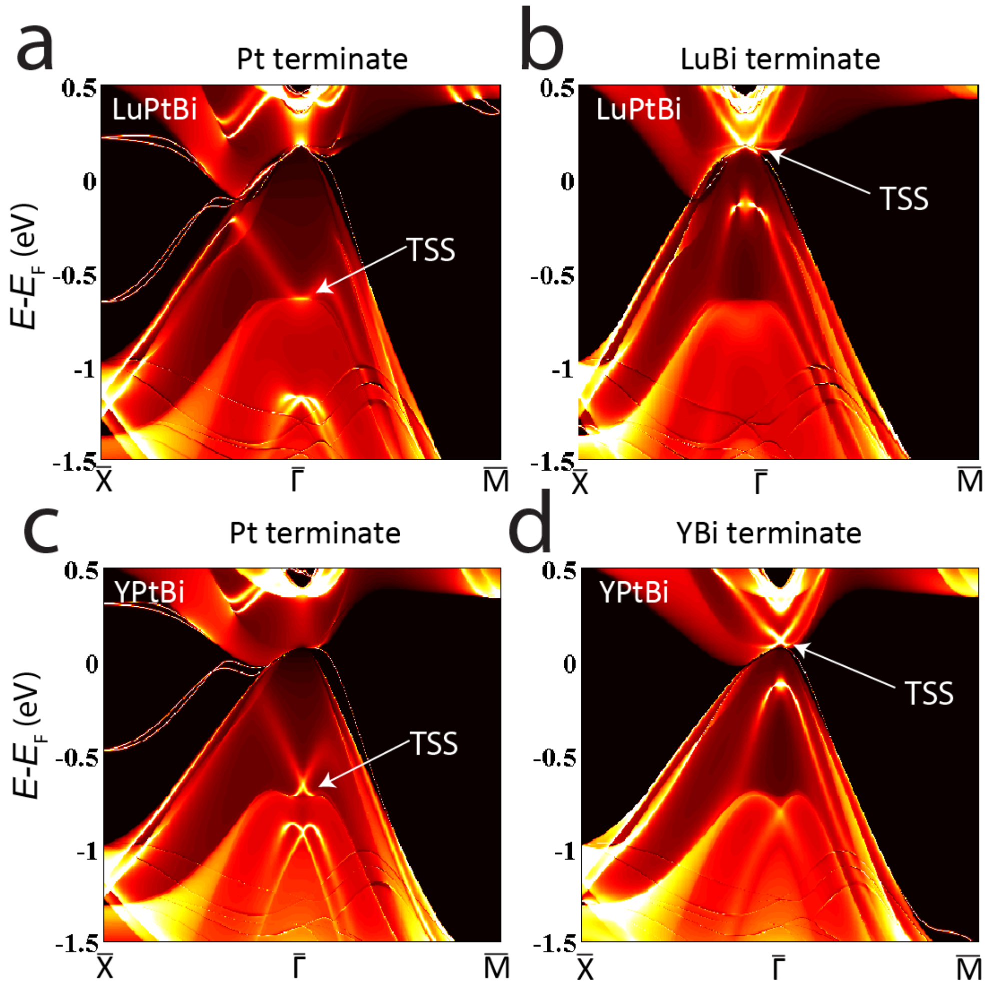

Supplementary Figure 7**: Ab-initio calculation of the electronic structure of (001) surface of LnPtBi with different terminations. a-b,** Calculation results from a semi-infinite surface using recursive Green's function on LuPtBi compound terminated by Pt (**a**) and Lu-Bi (**b**). **c-d**, Calculation results from a semi-infinite surface using recursive Green's function on YPtBi compound terminated by Pt (**a**) and Y-Bi (**b**). The metallic surface states due to surface dangling bonds are not captured by the calculation. TSS: topologically non-trivial surface state.

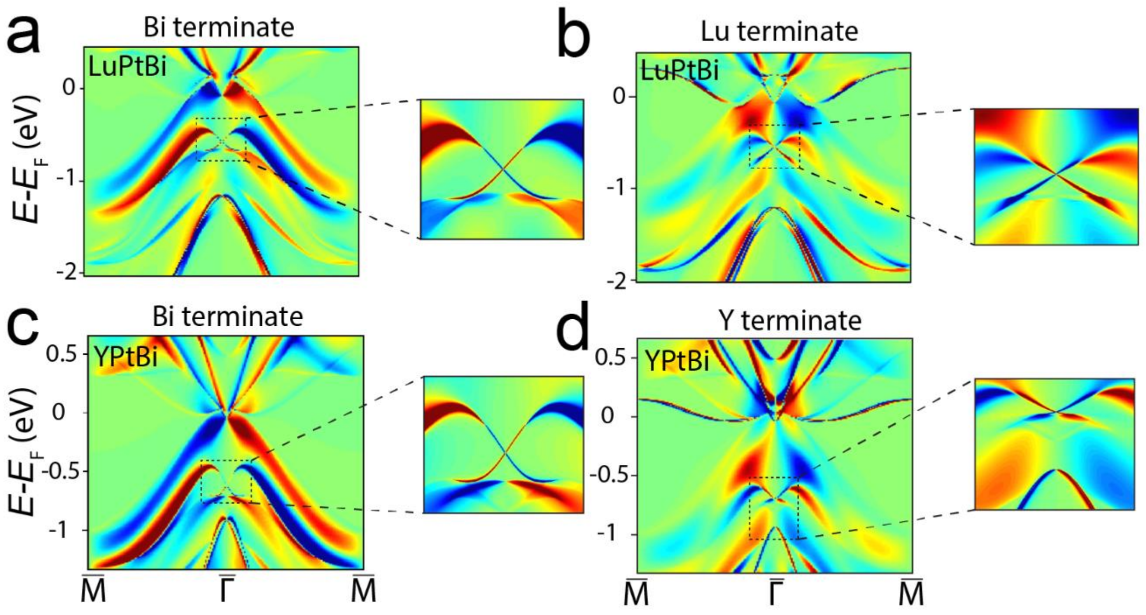

Supplementary Figure 8: **Ab-initio calculation results on spin-resolved bandstructure of the (111) surface of LuPtBi and YPtBi with different terminations. a-b**, Calculated spin-polarization of the bandstructure of the (111) surface of LuPtBi with Bi termination (**a**) and Lu termination (**b**). **c-d**, Calculated spin-polarization of the bandstructure of the (111) surface of YPtBi with Bi termination (**c**) and Y termination (**d**). All the insets show the zoomed-in plot of the 'X' shape band, which is the topologically non-trivial surface state.

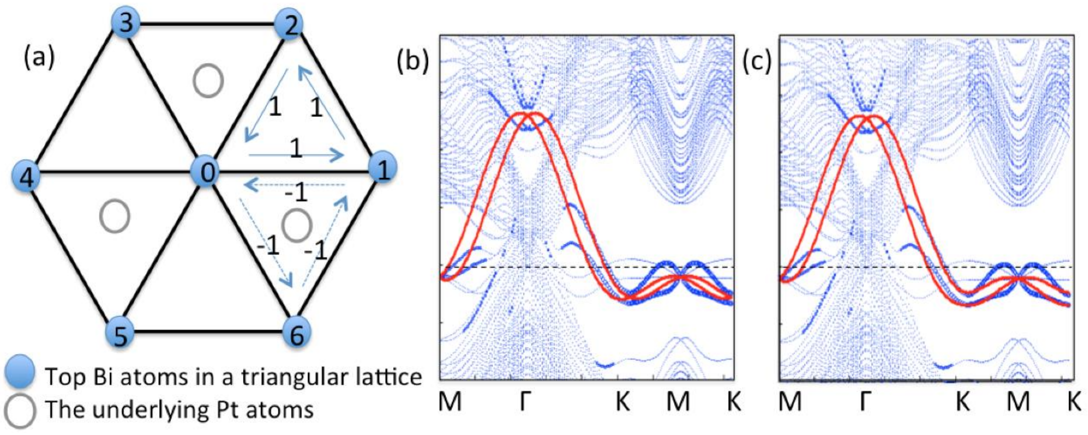

Supplementary Figure 9: **Tight-binding model of the triangular lattice of the Bi layer. a**, The triangular lattice. The blue circles represent the top Bi atoms and open circles represent the underlying Pt atoms. The ν_{ij} (±1) is labeled with respect to the hopping direction. **b-c**, The *ab-initio* surface band structures and the tight-binding fitting without and with the Kane-Mele term H^{KM}. The surface Bi-p_z projection is represented by the size of blue circles. The tight-binding fitted curves are in red color.

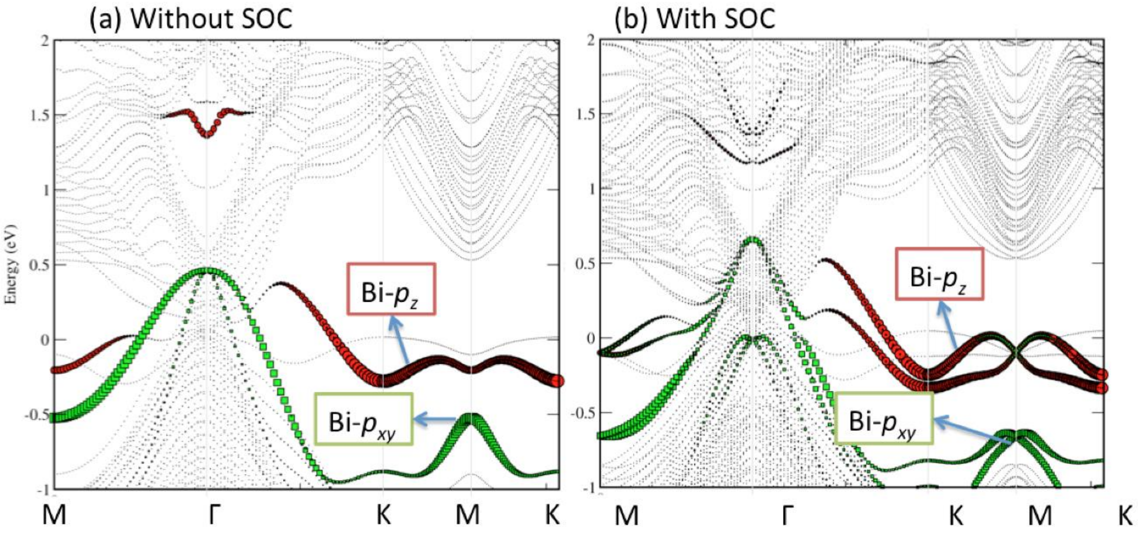

Supplementary Figure 10: ***Ab-initio* surface band structures of Bi-terminated (111) surface of LuPtBi without and with considering the SOC.** It is clear that the SS1 surface state is due to Bi-p_z orbital of the top layer, which is highlighted by red circles. The Bi-p_{xy} states are indicated by green circles.

Supplementary Note 1: Discussion on the Fermi crossings on the (111) surface of LuPtBi in the $\bar{\Gamma}$ vicinity

As we take a close look on the bandstructure of (111) surface of LuPtBi, we noticed there is a weak band near $\bar{\Gamma}$ next to the SS2 band (see Supplementary Figure 1**a** above, indicated by the green arrow). The position and shape of this band is similar to the band reported in Ref. 27, which was suggested in Ref. 27 as a Rashba type surface state. However, the fact that this band only shows sharp single branch dispersion makes it very different from other Kramers pair in this materials, such as SS2 and SS3 nearby that both show clear (and large) spin-splitting.

To investigate the origin of this band, we first carry out photon energies dependent measurement and study its k_z-dispersion (Supplementary Figure 1**b,c**). As clearly shown in Supplementary Figure 1**c**, the position of the Fermi surface crossing and shape does not show much variation along k_z, showing its surface origin. However, this band also shows considerable difference from other Rashba type surface states nearby, such as adjacent SS2 and SS3 bands in that: (1) this band does not come in pairs and (2) the dispersion has much weaker intensity comparing to SS3 and SS2 in ALL measurements. These differences indicate that this band is not typical Rashba split surface state (such as SS2 and SS3).

In order to understand the nature of this band, we carry out *ab-initio* calculations under different conditions. From Supplementary Figure 2**a-c**, the calculations show the evolution of the band dispersion projected to thin and thick (2-4 layers) surface slabs. While the SS2 and SS3 bands show similar intensity, the weak band (highlighted by the transparent red ovals in the zoom-in plot in Supplementary Figure 2**a-c**) under investigation shows clear difference: in two-layer slab, this band is very weak, but with the increase of the slab thicknesses, the spectra weight of this band clearly increases. Such enhancement of spectral weight with thicker slabs, together with its surface nature we demonstrated in Supplementary Figure 1, suggest that it is a surface resonant state (a

mixture of surface and bulk states), which satisfactorily explains the absence of the k_z dependence and its different features from Rashba type surface states (such as SS2 and SS3).

Supplementary Note 2: Further discussion on the hybridization of the TSS with bulk states on the (111) surface of LuPtBi

Since the topologically nontrivial surface states (TSS) do not locate in the bandgap but merge into the bulk valence band, it would show strong interaction with the bulk electronic states. As shown in the *ab-initio* calculation (Fig. 3b and Supplementary Note 6), the topological surface state indeed demonstrates strong hybridization at the tip point where the TSS bends back, characterized by the broadening of the spectral width (due to the reduction of the lifetime and k_z broadening effect of the bulk electronic states). Such broadening at the tip point is consistent with our observation (see Supplementary Figure 3**a**(ii),**b**(ii)).

To further confirm the hybridization between TSS and bulk states, in Supplementary Figure 3 we chose two momentum distribution curves (MDCs) that cut through the "X" shape dispersions at different binding energy to demonstrate their different k_z dispersions (MDC1 cuts through the back-bending part near the band top (E_b=0.41eV) and MDC2 cuts through the linear dispersion part (E_b=0.67eV), respectively).

For better illustration, we mark the MDC peaks related to back-bending part (blue circles, see Supplementary Figure 3**c**(i)), indeed they show clear variation with different photon energy. Such behavior shows clear difference from the k_z dispersion of MDC2 (which comes from the linearly dispersing part) where the green circles (indicating the MDC peaks, see Supplementary Figure 3**c**(ii)) show no photon energy dependent variation. The k_z dispersion shown in

Supplementary Figure 3 thus provides clear experimental evidence of the hybridization between the back-bending part of the TSS dispersion and the bulk state.

Supplementary Note 3: Photon energy dependent measurements on the dispersion along the $\bar{\Gamma}$-$\bar{M}$ direction on the (111) surface of LuPtBi

Similar to the Fig. 4(a) in the main text, we performed the photon energy dependent measurement along the $\bar{\Gamma}$-$\bar{M}$ direction on the Bi-terminated (111) surface of LuPtBi. And the result is plotted in Supplementary Figure 4. As we can see, neither the metallic surface states (SS1-SS3) nor the topological surface state (TSS) shows observable variation with position or shape when probed with different photon energy. Such observation once again establish the surface nature of SS1-SS3 and the TSS, consistent with the conclusion in the main text.

Supplementary Note 4: Photoemission and *ab-initio* calculation results on the (111) surface of YPtBi

The electronic structure of the (111) cleavage surface of YPtBi measured by ARPES is plotted in main text Fig. 4(e-g). From the Fermi surface map (Fig. 4(e)) and high symmetry $\bar{\Gamma}$-$\bar{K}$-$\bar{M}$ cuts ((Fig. 4(f-g))) we could identify several band dispersions distinct from the LuPtBi case (see Fig. 2-3). By measuring the bandstructure at several photon energies (not shown) and comparing with the theoretical calculations (Supplementary Figure 5), we conclude the feature around the E_F at the M point is the trivial surface state due to the dangling bond, while the band sitting ~700 meV below E_F around Γ is the topologically non-trivial surface state.

Notably, the measured electronic structures show better agreement with the *ab-initio* calculation results with Y termination (Supplementary Figure 5**c,d**). Combined with the results obtained in the main text, our observation of the topological surface state proved the validity of

the calculations with different materials and different terminations and showed the topological non-trivial surface state is the universal feature in LnPtBi (111) surfaces regardless of the termination.

Supplementary Note 5: Photoemission and *ab-initio* calculation results on the (001) surface of LnPtBi

The (111) surface is not the only natural cleavage surface of LnPtBi. At times we observed electronic bandstructure from LnPtBi (001) surfaces. Supplementary Figure 6**a** shows the unit-cell of LnPtBi (001) surface, which hosts a tetragonal surface BZ (Supplementary Figure 6**b**). Sample cleavage along the [001] direction breaks both Pt-Bi and Pt-Ln bonds and creates surfaces with terminating layers dominated by either Pt or Ln + Bi.

Supplementary Figure 6**c-e, f-h** shows the electronic structure from the (001) suface of YPtBi (**c-e**) and LuPtBi (**f-h**). Only features around $\bar{\Gamma}$ point are observed. In addition, no features similar to the topological surface states have been observed in either of the compounds.

We carried out *ab-initio* calculations with the Green's function method from the semi-infinite (001) surface of YPtBi and LuPtBi and found in both compounds, the topological surface state exists below the Fermi level in Pt terminated surface and above the Fermi level in Ln-Bi terminated surfaces (Supplementary Figure 7). As the measured bandstructure has a better agreement with the Ln-Bi terminated results, it is very likely the topological surface state is in the unoccupied states in the samples we have measured.

Supplementary Note 6: *Ab-initio* calculation results on spin-resolved bandstructure of *Ln*PtBi

To further prove the surface state at the $\bar{\Gamma}$ point is topologically nontrivial, we calculated the spin polarization of the bands from a semi-infinite surface and discovered (Supplementary Figure 8): (1) each branch of the $\bar{\Gamma}$ point surface state (the 'X' shape bands) is fully spin polarized with opposite direction. (2) The polarization of the surface state changed the sign at the tip of the 'X' shape where it hybridizes with other bulk states. The flip of the polarization proves the 'X' shape feature is not part of a Rashba-type surface state which would keep the same polarization in each of its parabolic branches. These observations prove the 'X' shape band is topologically non-trivial surface state with helical spin structure. We also carried out calculation on the (001) surface of LuPtBi and YPtBi and obtained similar results.
